# Muscle symptoms and creatine phosphokinase elevations in patients receiving raltegravir in clinical practice: results from a multicenter study

**DOI:** 10.1186/1758-2652-13-S4-P111

**Published:** 2010-11-08

**Authors:** G Madeddu, V Soddu, E Ricci, T Quirino, B Menzaghi, C Bellacosa, C Grosso, S Melzi, L Valsecchi, M Franzetti, F Vichi, G Penco, A Di Biagbio, G Pellicanò, L Corsico, GVL De Socio, E Mazzotta, G Parruti, M Guastavigna, G Orofino, MS Mura, P Bonfanti

**Affiliations:** 1 CISAI Group, Milan, Italy

## Purpose of the study

To further investigate CPK increases and muscle symptoms in a cohort of HIV-infected patients receiving raltegravir-based HAART compared with a control cohort with similar characteristics receiving darunavir-based HAART.

## Methods

The SCOLTA Project is a prospective, observational, multicenter study created to assess the incidence of adverse events in patients receiving new antiretroviral drugs in clinical practice. Muscle symptoms where classified according to the American Heart Association guidelines and CPK elevations were graded according to the DAIDS table.

## Results

A total of 391 HIV-infected patients were included in the study, 258 (66.0%) males. CDC stage was C in 152 (38.9%) patients. Mean age at enrolment was 44.5 ± 9.0 years, mean CD4 cell count 348 ± 260 cell/µL and mean HIV-RNA 3.26 ± 1.54 log10 cp/ml; 135 (35.2%) patients were HCV Ab+ and 155 (39.6%) had a diagnosis of lipodystrophy. Sixteen (4.1%) were naive to antiretrovirals. No statistical difference was observed regarding baseline characteristics when comparing patients receiving raltegravir (n=293) and darunavir-based HAART (n=98). Thirteen patients (5.4%) receiving raltegravir referred muscle pain and 12 (5.0%) muscle weakness compared with 1 (1.1%) and 0 (0%) receiving darunavir (p=0.20 and p=0.04), respectively. Seventeen (5.8%) patients developed muscle pain and/or weakness in the raltegravir cohort in respect of 1 (1.0%) in the darunavir cohort (p=0.05). No statistical difference was observed when considering CPK increases (>200 U/L) that were reported in 26 (8.9%) patients treated with raltegravir and 11 (11.2%) receiving darunavir. No relation emerged between CPK increase and muscle pain/weakness. Of note, no patient discontinued raltegravir due to CPK elevations or worsening muscle symptoms and no cases of rhabdomyolysis were reported.

## Conclusions

CPK elevations occurred in >10% of patients receiving both raltegravir and darunavir suggesting a multifactorial aetiology in HIV-infected patients treated with HAART. Raltegravir-treated patients had a significantly higher proportion of muscle symptoms, especially muscle weakness. However, these symptoms did not cause therapy discontinuation, in our cases. Although the limited clinical significance to date, our data suggest the monitoring of muscle symptoms, including both pain and weakness, in patients receiving raltegravir and further diagnostic evaluations if they persist.

